# Unveiling cholesterol metabolism-related gene ACOX2: a multi-omics discovery of a novel biomarker in IgA nephropathy

**DOI:** 10.1186/s41065-026-00639-0

**Published:** 2026-01-15

**Authors:** Xiaoqi Deng, Jinlan Wu, Mengxi He, Lin Mei, Li Ma, Yun Lin, Yu Luo

**Affiliations:** 1 Department of Nephrology, Zigong Fourth People’s Hospital, Zigong, 643000 Sichuan Province China; 2 Chongqing Medical University, Chongqing, 400000 China

**Keywords:** IgA nephropathy, ACOX2, Multi-omics, Biomarker, Cholesterol metabolism

## Abstract

**Background:**

The role of cholesterol metabolism in IgA nephropathy (IgAN) remains poorly understood.

**Methods:**

We applied a multi-omics integrative framework to systematically identify key regulatory genes. This approach combined genome-wide association study (GWAS), summary-data-based mendelian randomization (SMR), conventional MR, Bayesian colocalization, single-cell RNA sequencing (scRNA-seq), bulk transcriptome validation, molecular docking, and molecular dynamics simulations.

**Results:**

ACOX2 was identified as a protective hub gene. Genetic analyses revealed an inverse association between ACOX2 expression and IgAN risk (OR = 0.917, 95% CI: 0.879–0.957; PPH4 = 90.75%). scRNA-seq demonstrated the downregulation of ACOX2 in proximal tubular cells, which was further confirmed in external datasets. Molecular docking and molecular dynamics simulation suggested flavin adenine dinucleotide (FAD) as a potential therapeutic ligand targeting ACOX2.

**Conclusion:**

This study uncovers a cholesterol metabolism–related regulatory axis in IgAN, establishes ACOX2 as a protective biomarker, and highlights a therapeutically actionable pathway; it provides mechanistic insights and translational opportunities for biomarker development and drug discovery.

**Supplementary Information:**

The online version contains supplementary material available at 10.1186/s41065-026-00639-0.

## Key Points

1. This study investigates the role of cholesterol metabolism in the pathogenesis of IgA nephropathy (IgAN) using a multi-omics approach.
2. ACOX2 was identified as a key hub gene in IgAN, with lower expression in proximal tubular cells of patients with IgAN.
3. Mendelian randomization (MR) and colocalization analyses show a significant negative association between ACOX2 expression and IgAN risk.
4. Flavin adenine dinucleotide (FAD) was identified as a potential therapeutic compound for IgAN, showing strong binding to ACOX2.
5. ACOX2 may serve as a potential biomarker and therapeutic target for IgAN

**Supplementary Information:**

The online version contains supplementary material available at 10.1186/s41065-026-00639-0.

## Introduction

 IgA nephropathy (IgAN) is the most prevalent primary glomerulonephritis worldwide and a leading cause of end-stage renal disease (ESRD) [1, 2]. The hallmark pathological feature of IgAN is the deposition of aberrantly glycosylated IgA1 (Gd-IgA1) and its immune complexes in the glomerular mesangium, which initiates local inflammation and progressive kidney damage [3, 4]. Despite significant advances, the underlying mechanisms of IgAN remain incompletely understood. Current diagnostic practices rely on renal biopsy, an invasive procedure unsuitable for routine monitoring. Non-invasive biomarkers for early diagnosis, disease monitoring, and therapeutic evaluation are urgently needed. Identifying novel biomarkers is essential to advance precision medicine and personalized treatment strategies for IgAN, ultimately improving clinical outcomes.

Cholesterol metabolism is fundamental for cell membrane integrity, signal transduction, and the synthesis of bile acids and hormones [5]. Dysregulation of cholesterol metabolism has been implicated in a range of pathological conditions, including cardiovascular diseases [6], immune system disorders [7], neurodegenerative diseases [8], and cancer [9]. Recently, its role in kidney diseases has gained increasing attention [10]. Emerging evidence suggests that cholesterol and its derivatives may exacerbate kidney inflammation and fibrosis by activating inflammatory and immune signaling pathways [11].

Cholesterol metabolism plays a critical role in cellular homeostasis, including membrane integrity, signal transduction, and immune regulation; the dysregulation of cholesterol metabolism has been implicated in multiple diseases. However, its specific role in IgAN remains largely unexplored. With advances in bioinformatics, many studies have used computational approaches to identify potential biomarkers. For example, bioinformatics has been applied to study hypoxia-associated genes in chronic obstructive pulmonary disease [12], to identify genes involved in lung cancer progression in idiopathic pulmonary fibrosis [13], and to uncover neuroinflammation-related hub genes in primary open-angle glaucoma [14]. Therefore, in this study, we aimed to integrate multiple bioinformatics approaches to investigate the role of cholesterol metabolism in IgAN.

**Fig. 1:**
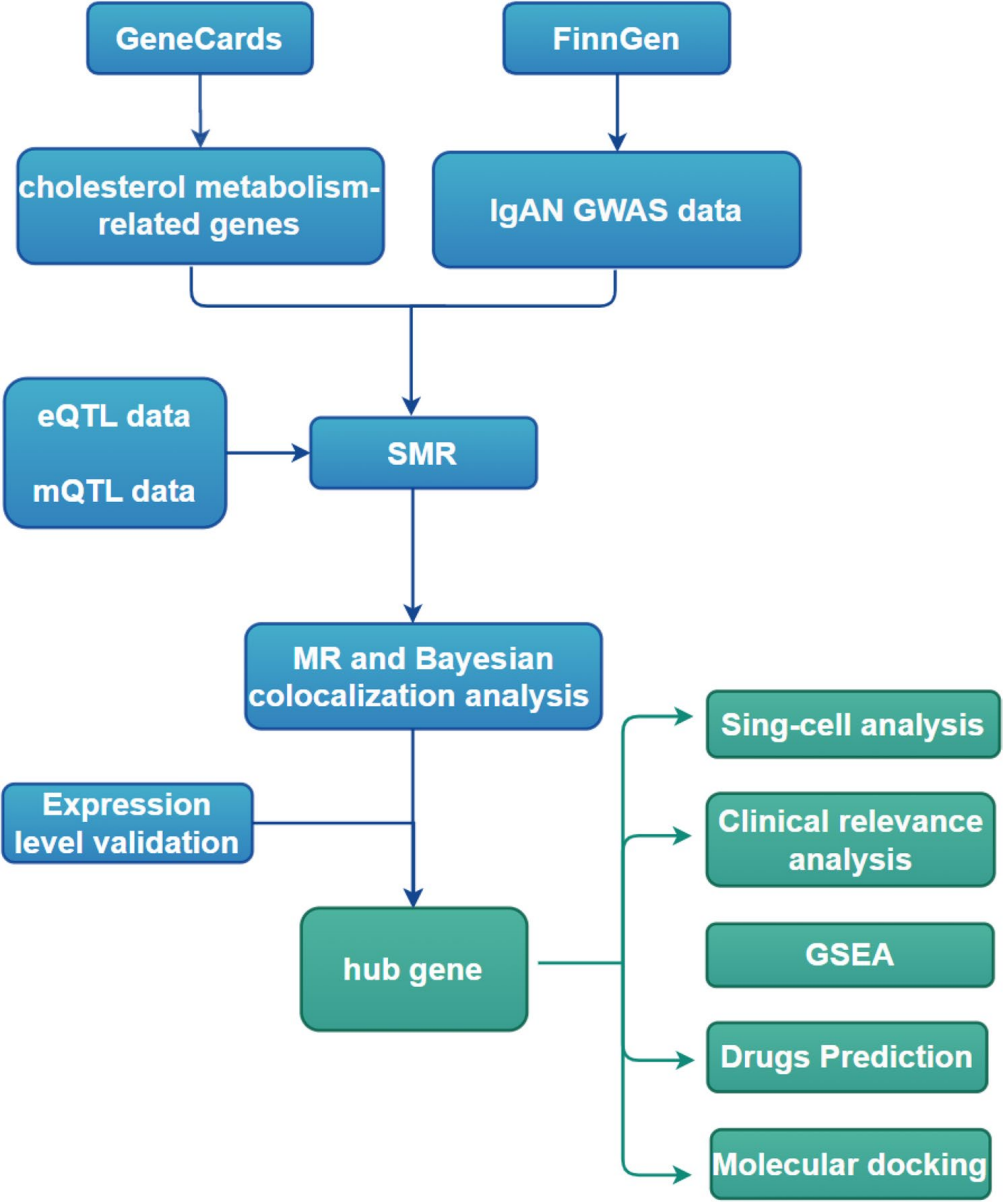
The flow chart of our study

This study investigates whether genetically determined alterations in cholesterol metabolism influence IgAN susceptibility and progression, with cholesterol metabolism-related genes as the exposure and IgAN risk as the outcome. Using Mendelian randomization (MR), which estimates causal effects under the assumptions of relevance, independence, and exclusion restriction, we integrate summary-data-based Mendelian randomization (SMR), conventional MR, colocalization, and single-cell transcriptomics to uncover molecular mechanisms and identify potential biomarkers for early diagnosis and prognosis. Furthermore, molecular docking and molecular dynamics simulations were performed to validate the predicted interactions and assess the structural stability of protein–ligand complexes. The detailed workflow is shown in Fig. 1.

## Materials and methods

### Data collection

Cholesterol metabolism-related genes were curated from the GeneCards database (https://www.genecards.org/) by querying the term “cholesterol metabolism” and applying a relevance score threshold of ≥ 10 to ensure high specificity. Genome-wide association study (GWAS) data for IgAN were obtained from the FinnGen study [15] (https://www.finngen.fi/), comprising 726 IgAN cases and 453,007 healthy controls (HCs). Blood cis-expression quantitative trait locus (cis-eQTL) summary statistics for cholesterol metabolism genes were sourced from eQTLGen, integrating cis-eQTL data for blood gene expression across 31,684 individuals pooled from 37 independent datasets [16]. Blood cis-methylation quantitative trait locus (cis-mQTL) summary statistics were generated via a meta-analysis of two cohorts: the Lothian Birth Cohorts (*n* = 1366) and the Brisbane Systems Genetics Study (*n* = 614) [17]. For external validation, IgAN GWAS summary statistics were retrieved from the GWAS Catalog (https://www.ebi.ac.uk/gwas/), encompassing 15,658 IgAN cases and 637,485 HCs, while GSE93798 and GSE127136 were accessed from the GEO database (https://www.ncbi.nlm.nih.gov/geo/).

### Summary-data-based MR and HEIDI analysis

SMR is a Mendelian randomization method based on data aggregation, which is used to elucidate pleiotropic relationships between genetic traits, such as gene expression, DNA methylation, or protein abundance, and complex traits, such as disease phenotypes, by integrating data from GWAS and QTL studies within the MR framework [18, 19]. We applied summary-data-based Mendelian randomization (SMR, version 1.3.1) to evaluate the potential causal relationship between cholesterol metabolism–related gene expression (exposures) and IgAN (outcome). Statistical significance was determined using the following criteria: *P*_FDR_ < 0.05 for SMR and *P*_HEIDI_ > 0.05 for the HEIDI test, where the latter ensures that associations are not confounded by linkage disequilibrium [20].

### MR and Bayesian colocalization analysis

MR analysis was employed as a supplementary method to validate whether the genes identified by SMR were causally associated with IgAN. This analysis was conducted using the “TwoSampleMR” R package [21], applying the inverse variance weighted (IVW) method with single-nucleotide polymorphisms (SNPs) as instrumental variables (IVs). The analysis explicitly assumes the three core IV conditions: relevance (SNPs are associated with the exposure), independence (SNPs are independent of confounders), and exclusion restriction (SNPs affect IgAN only via the exposure). Sensitivity analyses, including MR-Egger and weighted median methods, were conducted under the same assumptions to assess potential heterogeneity and pleiotropy. Additionally, Bayesian colocalization analysis was carried out to assess whether the observed gene expression-IgAN associations were attributable to shared causal variants. This analysis was performed using the “coloc” R package [22], with posterior probability 4 (PPH4) ≥ 0.8 considered indicative of strong colocalization.

### Gene expression level validation of hub genes

To validate the gene expression levels of hub genes between IgAN and the healthy group, the GSE93798 dataset was retrieved from the GEO database, and a t-test was performed, followed by the plotting of receiver operating characteristic (ROC) curves.

### Single-cell analysis

The RNA-seq dataset GSE127136, comprising 26,398 genes from 18,350 cells derived from IgAN tissue, was obtained from the GEO database. The 10x gene expression matrices were imported and processed using the “Seurat” R package [23]. Quality control procedures were applied to exclude low-quality cells, ensuring data reliability. The data were normalized using the LogNormalize method, and highly variable genes were identified for subsequent dimensionality reduction. Principal component analysis (PCA) was performed to reduce dimensionality, and Uniform Manifold Approximation and Projection (UMAP) was utilized to visualize the structural relationships among cell populations. Clustering analysis was conducted using the FindNeighbors and FindClusters functions, with UMAP providing cluster visualization. To address potential batch effects, the Harmony algorithm was applied for batch correction, enhancing the robustness of the analysis. Cell type annotation was performed based on the expression of marker genes, and the proportions of each cell type were calculated for different groups. Differential expression analysis was conducted to identify significant gene expression changes, which were subsequently visualized for interpretation. Differential expression analysis was conducted using Seurat’s Wilcoxon rank-sum test, followed by Benjamini–Hochberg false discovery rate (FDR) correction. Genes with an adjusted *P* value < 0.05 were considered statistically significant and were subsequently visualized for interpretation.

### Clinical relevance analysis

The Nephroseq V5 database [24] (https://v5.nephroseq.org/) is an online platform that provides comprehensive data on kidney-related conditions, including gene expression profiles, clinical characteristics, and patient outcomes for a range of kidney diseases, such as IgA nephropathy, diabetic nephropathy, and acute kidney injury. In this study, Nephroseq V5 was used to investigate the association between hub genes and key clinical features of IgAN.

### Gene set enrichment analysis

Gene Set Enrichment Analysis (GSEA) is a widely employed method to assess the enrichment of predefined gene sets across different phenotypes or conditions [25]. By analyzing the ranked gene expression data and comparing it with the distribution of specific gene sets, GSEA identifies the activation or inhibition of associated biological processes and signaling pathways. In this study, we first calculated the Pearson correlation coefficients between ACOX2 and all genes in the IgAN samples of the GSE93798 dataset. Subsequently, we performed GSEA based on these correlation coefficients to explore the potential functions of ACOX2. To ensure rigorous control of multiple testing, the Benjamini–Hochberg false discovery rate (FDR) correction was applied, and gene sets with an adjusted *P* value (FDR q-value) < 0.05 were considered statistically significant.

### Potential drug prediction and molecular docking

The Bioinformatics Annotation Database for Molecular Mechanisms of Traditional Chinese Medicine [26] (BATMAN-TCM 2.0, https://bionet.ncpsb.org/) was utilized to predict potential therapeutic drugs based on the hub genes. Molecular docking was performed using CB-Dock2 [27] (https://cadd.labshare.cn/cb-dock2/) to assess the interaction between the hub genes and drugs.

### MD simulation

MD simulations were performed using GROMACS 2023.2 [28], with ligand topologies generated by AMBER/ACPYPE and protein topologies by CHARMM36. Systems were solvated with TIP3P water, neutralized with Na⁺/Cl⁻ ions, and simulated for 100 ns at 300 K and 1 bar with 2 fs time steps under three-dimensional periodic boundary conditions. Hydrogen bonds were constrained using LINCS; short-range interactions were truncated at 1.2 nm, and long-range electrostatics were treated with PME.

### Quantitative real-time PCR

Peripheral blood samples from patients with IgA nephropathy (IgAN, *n* = 8) and healthy controls (*n* = 8) were collected from Zigong Fourth People’s Hospital for quantitative real-time PCR validation of key gene expression. Total RNA was extracted from samples using TRIzol reagent (GPLBIO, Cat# GK20008) following standard phenol–chloroform extraction procedures. RNA concentration was determined using a NanoDrop spectrophotometer, and 1 µg of total RNA was reverse-transcribed into cDNA using a reverse transcription kit (TAKARA, RR047A) after genomic DNA removal according to the manufacturer’s instructions. The resulting cDNA was diluted 3–5 fold and used for quantitative real-time PCR, which was performed with SYBR Green Master Mix (ABclonal, Cat# RK21203, China) on a LightCycler^®^ 480 Real-Time PCR System (Roche). Relative gene expression levels were calculated using the 2^−ΔΔCt method with GAPDH as the internal reference. All reactions were performed in triplicate. Primer sequences were as follows: ACOX2 (forward, 5′-GCACCCCGACATAGAGAGC-3′; reverse, 5′-CTGCGGAGTGCAGTGTTCT-3′) and GAPDH (forward, 5′-GGAGCGAGATCCCTCCAAAAT-3′; reverse, 5′-GGCTGTTGTCATACTTCTCATGG-3′).

## Results

### Identification of important genes

**Fig. 2:**
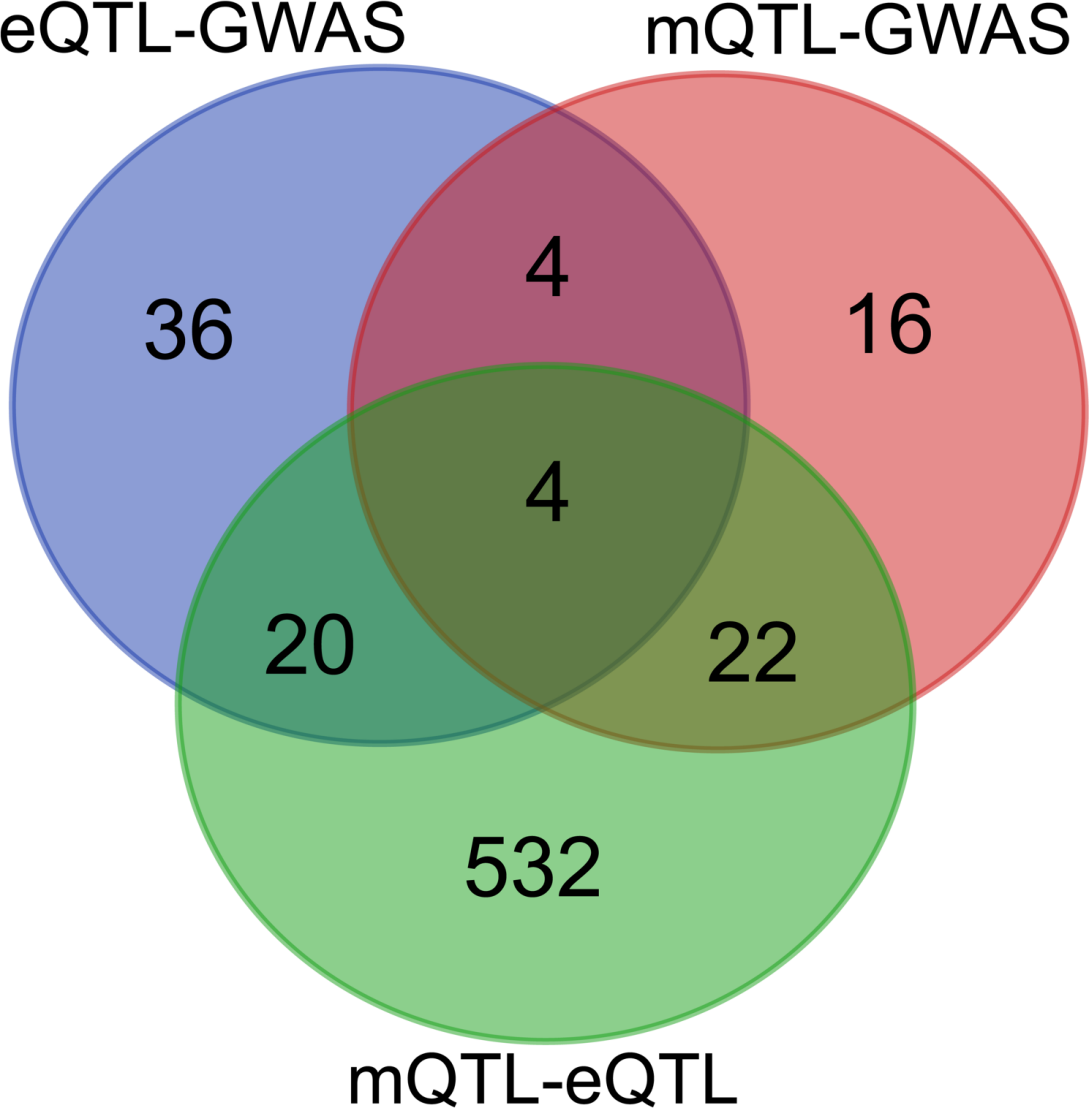
The identification of 4 key genes among eQTL-GWAS, mQTL-GWAS, and mQTL-eQTL. From this integration, eQTL-GWAS identified 64 genes, mQTL-GWAS identified 46 genes, and further integration of cis-eQTL and cis-mQTL data (mQTL-eQTL) prioritized 578 genes, ultimately leading to the identification of 4 key genes (ACOX2, ADAR, HPD, and MBTPS1) associated with IgAN risk

A total of 1,645 cholesterol metabolism-related genes, with relevance scores ≥ 10, were retrieved from the GeneCards database (Table S1). Their cis-eQTLs (*n* = 2,185,958 SNPs) and cis-mQTLs (*n* = 3,904,899 CpG sites) were subsequently integrated with GWAS summary datasets for IgAN. From this integration, 64 cholesterol metabolism-related genes were identified through the eQTL-GWAS analysis (*P*_FDR_ < 0.05, *P*_HEIDI_ > 0.05, Table S2), while 108 DNA methylation (DNAm) probes (representing 46 genes within 1 Mb) were selected via the mQTL-GWAS integration (*P*_FDR_ < 0.05, *P*_HEIDI_ > 0.05, Table S3). Further integration of cis-eQTL and cis-mQTL data prioritized 2,114 DNAm probes (578 genes within 1 Mb, *P*_FDR_ < 0.05, *P*_HEIDI_ > 0.05, Table S4). By intersecting the results from these analyses, four significant genes—ACOX2, ADAR, HPD, and MBTPS1—were identified (Fig. 2).

**Fig. 3:**
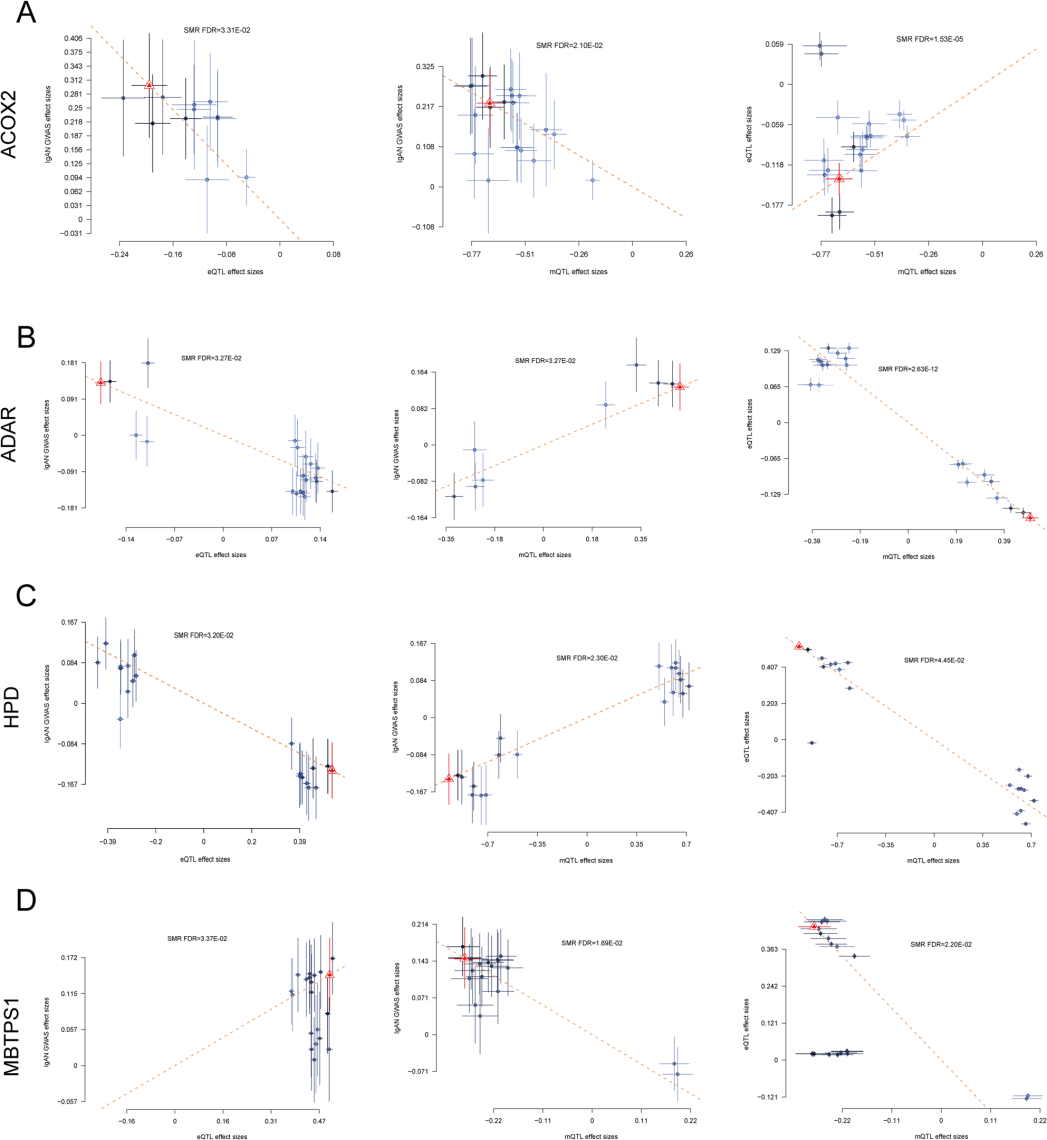
The association between the expression levels of four key genes, IgAN, and DNA methylation (DNAm) levels. **A** The DNAm probe cg16209444 exhibited a positive correlation with ACOX2 expression. Higher expression levels of ACOX2, along with increased methylation, were associated with potentially reduce the risk of IgAN. **B**-**C** The DNAm probes cg27530370 and cg08980965 were negatively correlated with the expression of ADAR and HPD, respectively. Lower methylation levels, accompanied by increased expression of ADAR and HPD, were also associated with a potential reduction in IgAN risk. **D** The DNAm probe cg26868097 showed a negative association with MBTPS1 expression and IgAN risk. Elevated MBTPS1 expression was positively correlated with an increased risk of IgAN. Analyses performed using Pearson correlation with FDR-adjusted *P* < 0.05

In Fig. 3A, the DNAm probe cg16209444 was positively associated with ACOX2 expression (beta_SMR_ = 0.035), while higher ACOX2 gene expression (beta_SMR_ = − 1.557) and increased methylation levels (beta_SMR_ = − 0.332) were associated with a potentially reduce the risk of IgAN. Figure 3B and C show that the DNAm probes cg27530370 and cg08980965 were negatively associated with ADAR (beta_SMR_ = − 0.682) and HPD (beta_SMR_ = − 0.537) expression, respectively. The increased expression of ADAR and HPD (beta_SMR_ = − 0.757 and beta_SMR_ = − 0.265), along with lower methylation levels (beta_SMR_ = 0.267 and beta_SMR_ = 0.142), were also found to potentially reduce the risk of IgAN. In Fig. 3D, the DNAm probe cg26868097 was negatively associated with MBTPS1 expression (beta_SMR_ = − 1.535) and IgAN risk (beta_SMR_ = − 0.521), while MBTPS1 expression was positively correlated with IgAN risk (beta_SMR_ = 0.289).

### MR and Bayesian colocalization analysis

To further validate the causal relationships between the above key genes and IgAN, we obtained external GWAS data for IgAN from the GWAS Catalog and performed MR and Bayesian colocalization analyses. The results revealed that SNPs associated with ACOX2 were linked to a reduced risk of IgAN (OR = 0.917, 95% CI: 0.879–0.957, *P* = 7.27E-05, SNP.PP.H4 = 90.75%), while SNPs associated with MBTPS1 were associated with an increased risk of IgAN (OR = 1.012, 95% CI: 1.005–1.020, *P* = 1.62E-03, SNP.PP.H4 = 99.92%). Similarly, SNPs associated with ADAR were correlated with a reduced risk of IgAN (OR = 0.98, 95% CI: 0.958–1.003, *P* = 7.87E-02, SNP.PP.H4 = 37.35%), whereas SNPs associated with HPD were linked to an increased risk of IgAN (OR = 1.006, 95% CI: 0.999–1.013, *P* = 7.26E-02, SNP.PP.H4 = 79.87%). Detailed information, including the results of heterogeneity and pleiotropy tests from the sensitivity analyses, is presented in Table 1.

**Table 1:** The results of the IVW MR and colocalization analysis

Exposure	Outcome	OR	95%CI	*P*-value	Pleiotropy *p*-value	Heterogeneity *p*-value	SNP.PP.H4
ACOX2	IgAN	0.917	0.879–0.957	7.27E-05	4.14E-04	1	90.75%
ADAR	IgAN	0.98	0.958–1.003	8.70E-02	1.96E-02	9.92E-01	37.35%
HPD	IgAN	1.006	0.999–1.013	7.26E-02	8.88E-01	1.98E-01	79.87%
MBTPS1	IgAN	1.012	1.005–1.020	1.62E-03	5.36E-04	9.29E-01	99.92%

### Identification of hub gene ACOX2

**Fig. 4:**
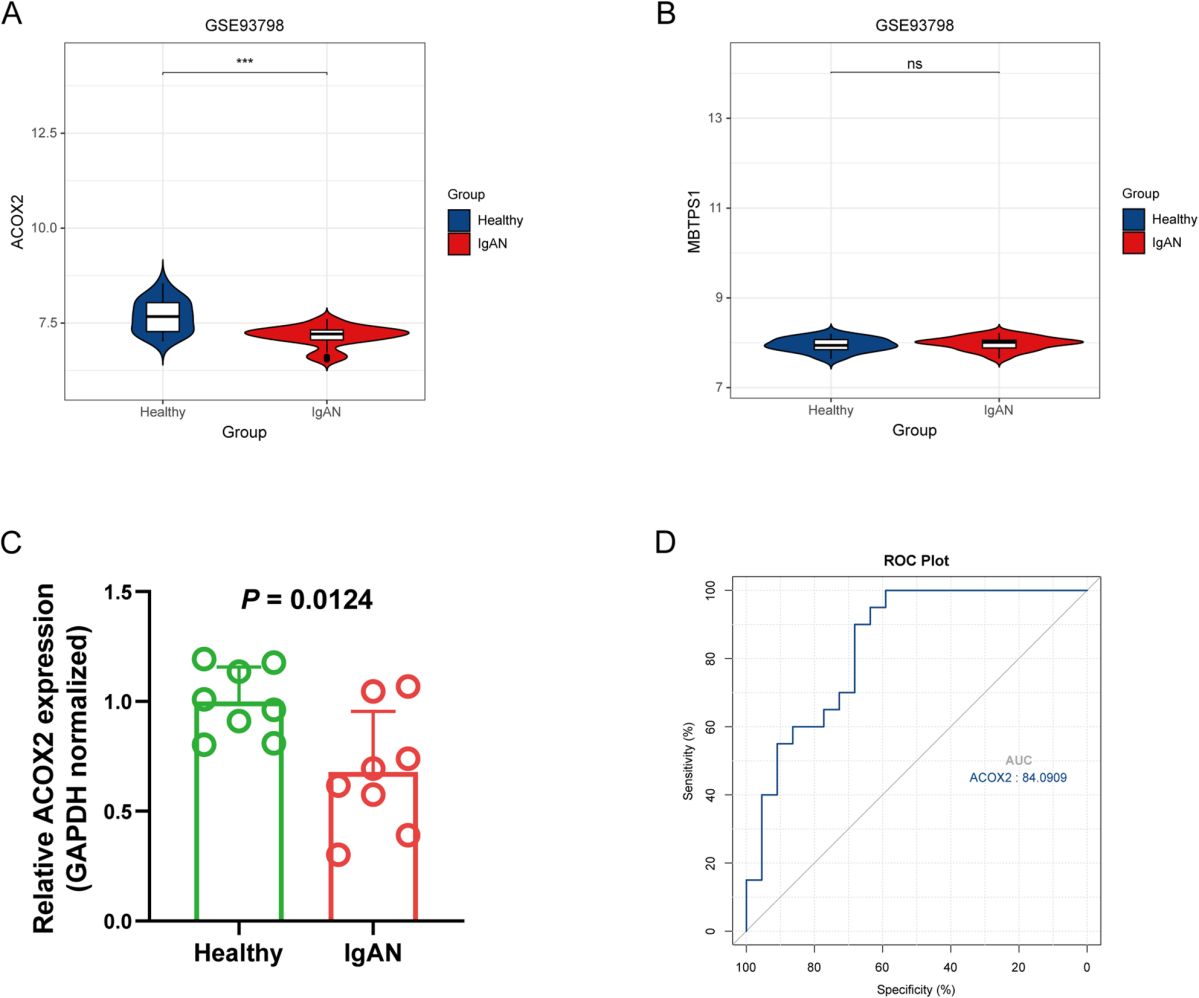
Identification of hub gene ACOX2. **A** ACOX2 expression was significantly lower in the IgAN group compared to the healthy controls group (*P* < 0.05, *n* = 20 IgAN vs. *n* = 22 healthy controls). **B** MBTPS1 expression was higher in the IgAN group than in the healthy group, but the difference was not statistically significant (*P* > 0.05). **C** The qRT-PCR validation of ACOX2 gene expression: ACOX2 expression was significantly lower in the IgAN group compared to the healthy group (*P* < 0.05). **D** The area under the curve (AUC) for ACOX2 was greater than 0.8, indicating its strong potential as a biomarker

**Fig. 5:**
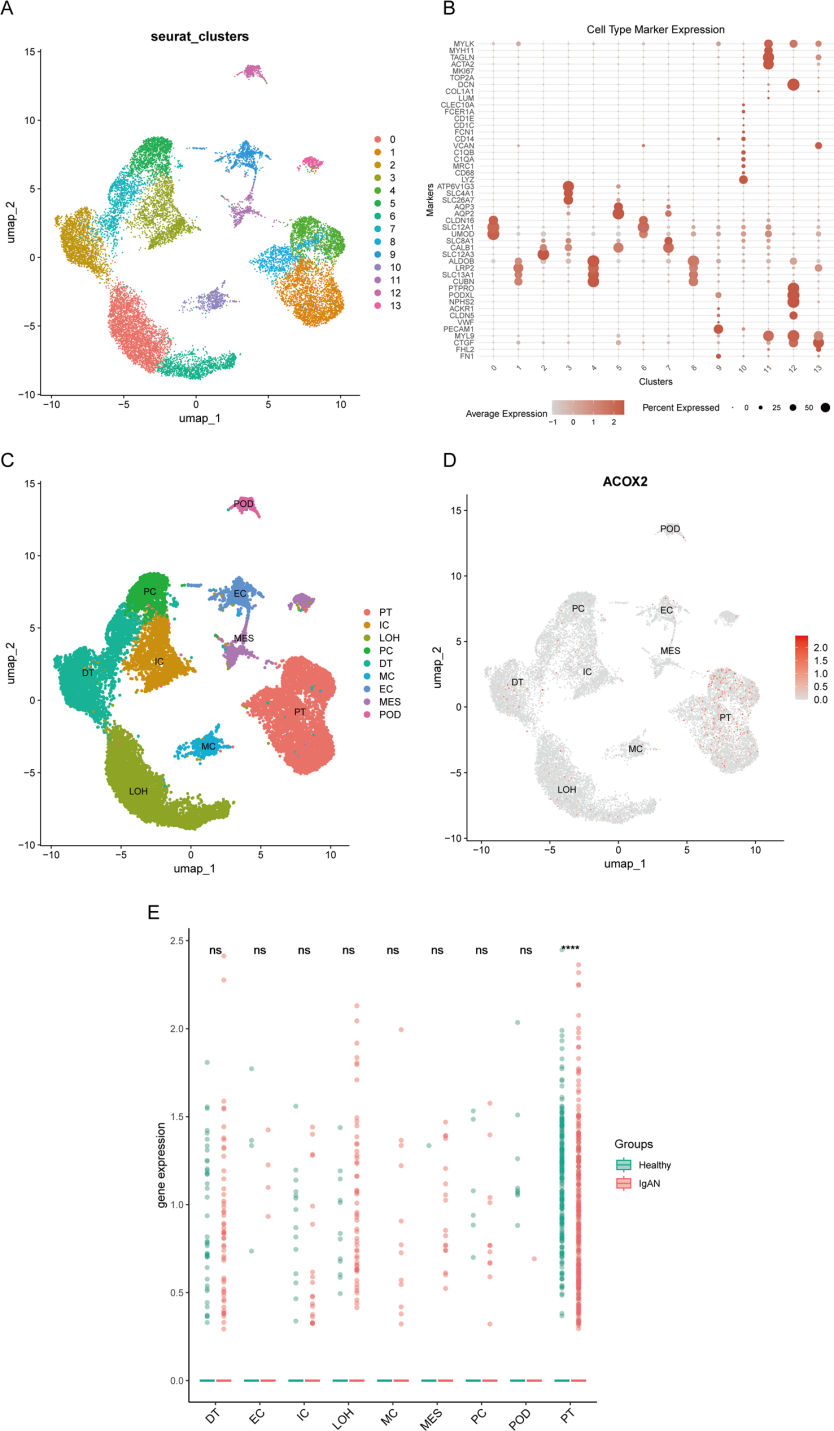
Single-cell analysis of ACOX2 in GSE127136. **A** UMAP plot of 13 identified cell clusters (*n* = 18,350 cells; GSE127136). **B** Cell type annotation corresponding to the identified clusters. **C** UMAP plot detailing the specific cells within the dataset. **D** ACOX2 expression is primarily observed in proximal tubular (PT) cells. **E** Differential expression in PT cells between IgAN and healthy controls (adjusted *P* < 0.05, Wilcoxon rank-sum test with Benjamini–Hochberg FDR correction). Data normalized using Seurat’s LogNormalize

To further validate the expression levels of ACOX2 and MBTPS1 in IgAN, the GSE93798 dataset was retrieved from the GEO database. A *t* -test analysis revealed that ACOX2 was significantly downregulated in the IgAN group compared to healthy controls (*P* < 0.05), whereas MBTPS1 showed an upregulation trend in the IgAN group, though not statistically significant (*P* > 0.05). Consistently, the results of qRT-PCR showed that the mRNA expression level of ACOX2 was significantly lower in the IgAN group than in the healthy control group (*P* < 0.05, Fig. 4C). The ROC curve was generated for ACOX2, yielding areas under the curve (AUC) exceeding 0.8 (Fig. 4D), highlighting its potential as a novel biomarker for IgAN. To further explore the expression profile of ACOX2, single-cell RNA sequencing analysis was performed. The results demonstrated that ACOX2 was predominantly differentially expressed in proximal tubular cells (PT) between IgAN and healthy groups (Fig. 5), suggesting its potential involvement in tubular dysfunction and its relevance to IgAN pathogenesis. These findings underscore the need for further studies to investigate the functional role of ACOX2 in IgAN.

### Clinical relevance and gene set enrichment analyses

**Fig. 6:**
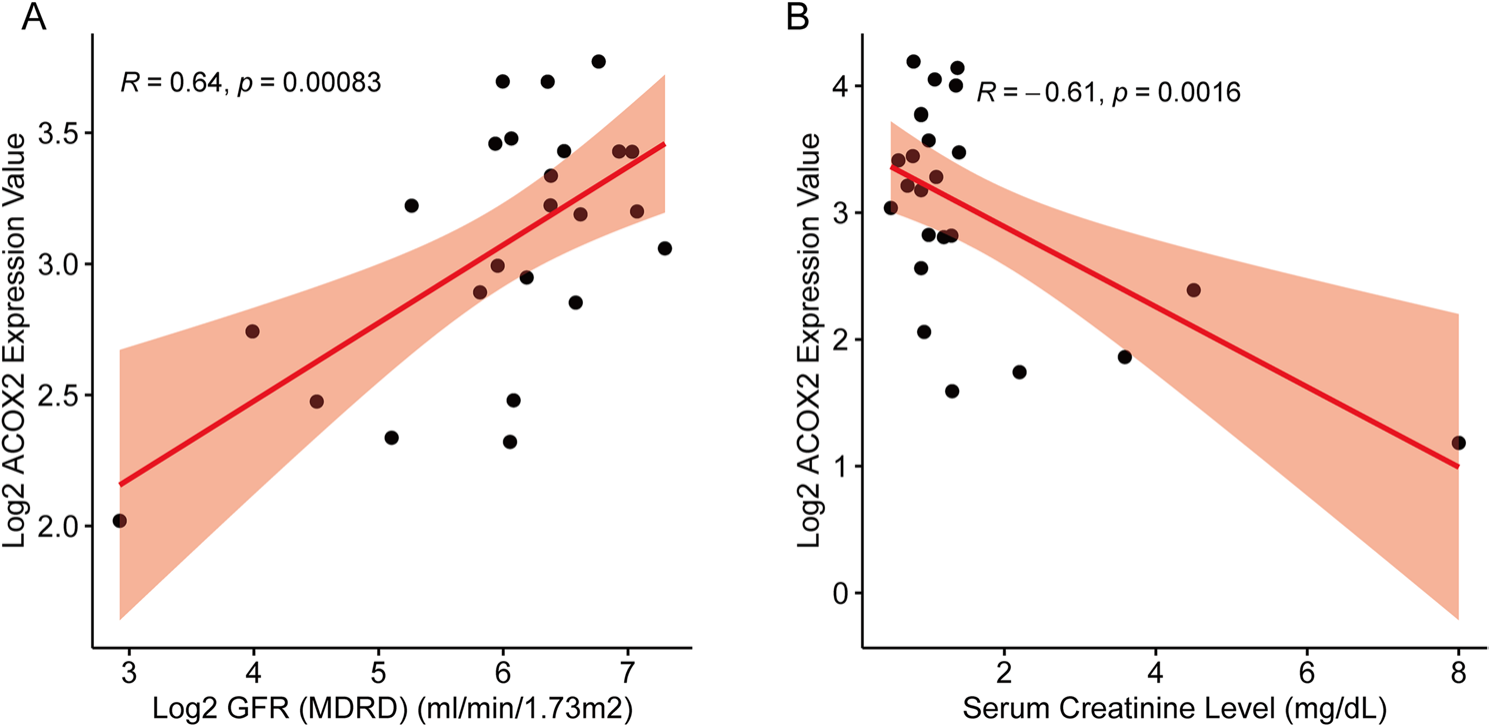
Clinical correlation analysis of ACOX2. **A** The expression level of ACOX2 was positively correlated with glomerular filtration rate (*R* = 0.64, *P* = 0.00083). **B** The expression level of ACOX2 was negatively correlated with serum creatinine levels *(R* = − 0.61, *P* = 0.0016)

**Fig. 7:**
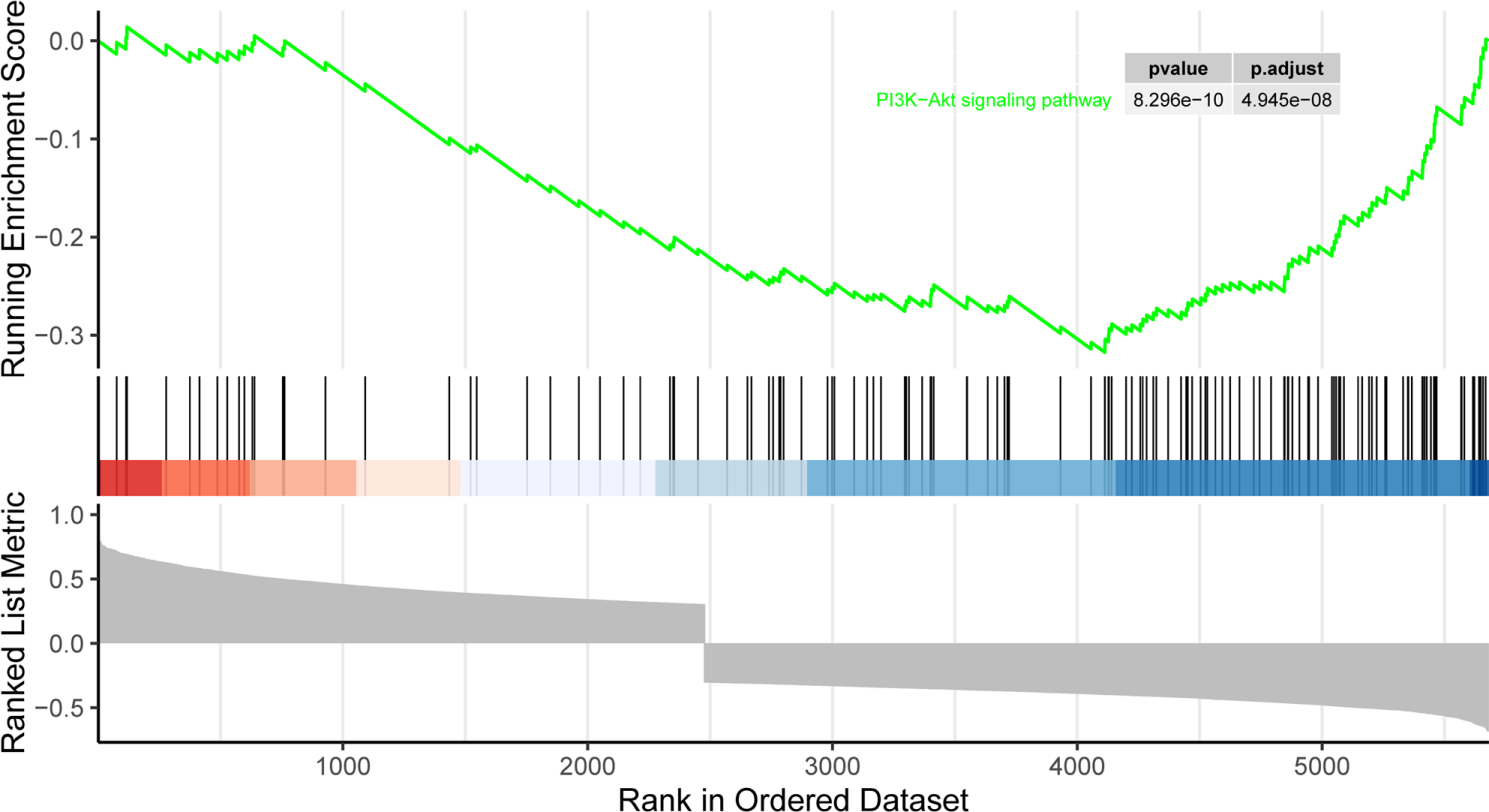
The result of GSEA. The ACOX2 was primarily enriched in the PI3K-Akt signaling pathway (adjusted *P* < 0.05)

To further confirm the relationship between ACOX2 and IgAN, we performed clinical correlation analysis and gene set enrichment analysis (GSEA). The clinical correlation analysis revealed an inverse association between ACOX2 expression and serum creatinine levels, along with a positive correlation with glomerular filtration rate (GFR), as shown in Fig. 6. GSEA indicated that ACOX2 was primarily enriched in the PI3K-Akt signaling pathway (Fig. 7), suggesting its potential involvement in key molecular processes that regulate cell survival, growth, and metabolism. Collectively, these results provide additional insights into the functional role of ACOX2 in IgAN and its potential as a therapeutic target for modulating kidney function.

### Drug prediction and molecular docking

**Fig. 8:**
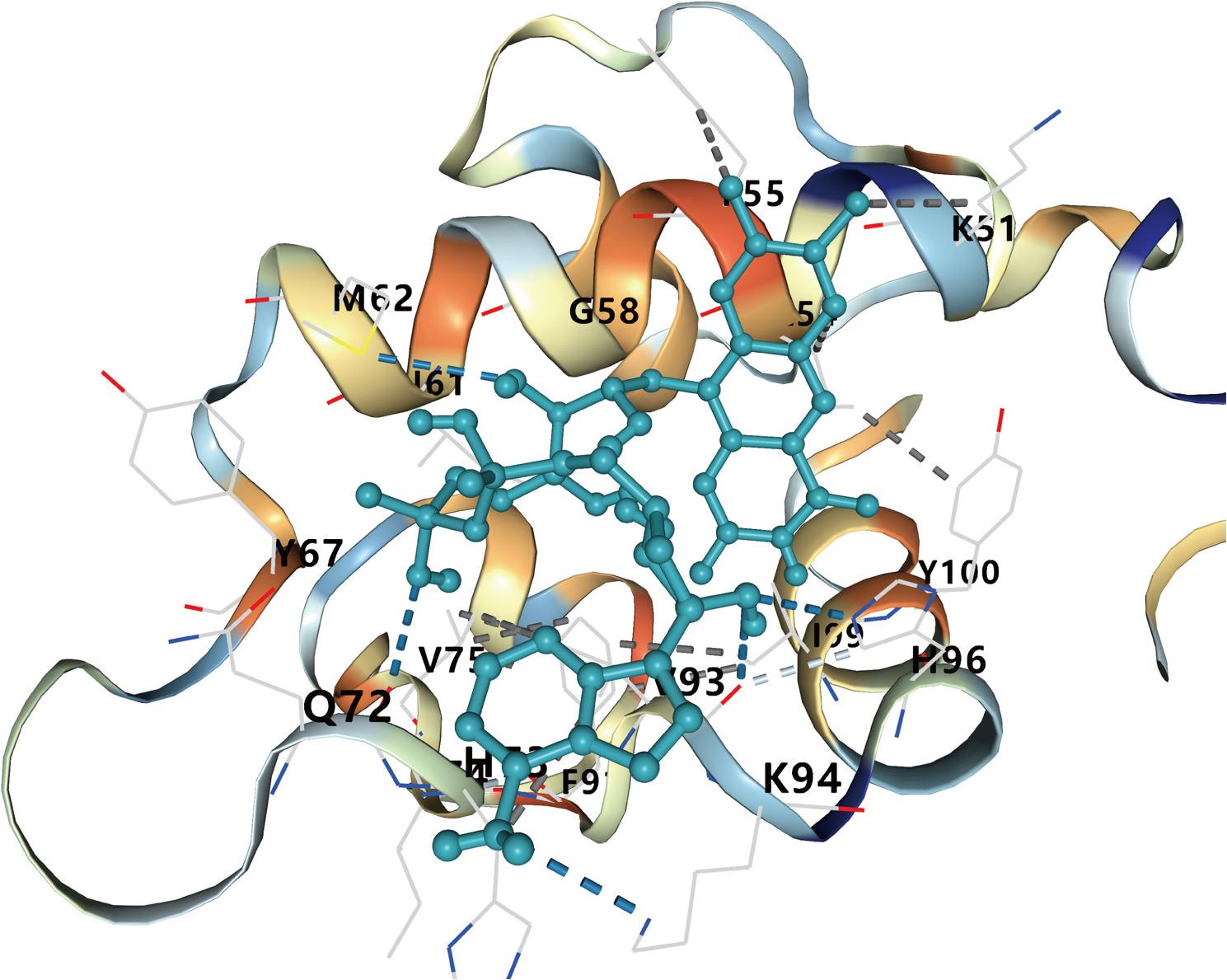
The result plot of molecular docking. The molecular docking analysis demonstrated that multiple amino acid residues in ACOX2 form stable hydrogen-bond interactions with flavin adenine dinucleotide (FAD). All calculated binding free energies for these complexes are below − 5 kcal/mol, indicating strong spontaneous binding affinity between ACOX2 and its FAD cofactor

Based on the identification of ACOX2, we utilized BATMAN-TCM to predict potential therapeutic agents. The results identified flavin adenine dinucleotide as a promising pharmacological target for IgAN. Molecular docking simulations between ACOX2 and flavin adenine dinucleotide revealed consistently favorable binding free energies below − 5.0 kcal/mol, supporting the potential interaction (Table 2; Fig. 8).

**Table 2:** The results of potential drug and module Docking

Gene	Drug	Confidence score	Likelihood ratio	Chemical Formula	Binding energy (kcal/mol)
ACOX2	flavin adenine dinucleotide	0.91	133.12	C_27_H_33_N_9_O_15_P_2_	-6.5

### Structural and energetic stability of the ACOX2–FAD complex

**Fig. 9:**
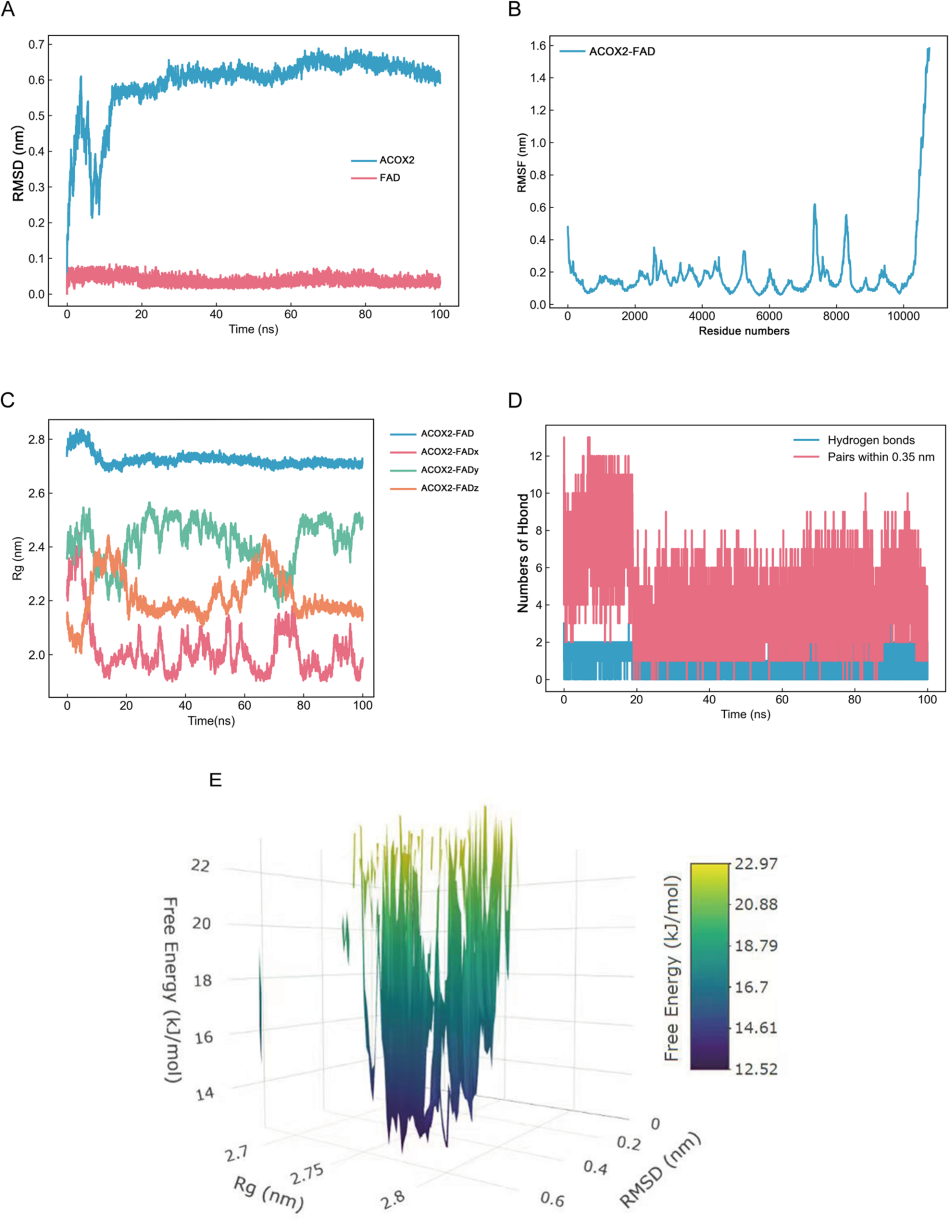
Molecular dynamics analysis of the ACOX2–FAD complex. **A** Time evolution of RMSD for the ACOX2 backbone (black) and FAD ligand (red). **B** The RMSF plot of ACOX2–FAD complex. **C** The Rg plot of the ACOX2–FAD complex. **D** Time-dependent count of intermolecular hydrogen bonds between ACOX2 and FAD, averaging 4–5 stable bonds that support binding stability. **E** Three-dimensional free energy landscape projected onto ligand RMSD, protein Rg, and Free energy

To assess the stability of the ACOX2–FAD complex, a 100-ns molecular dynamics simulation was performed. The protein backbone RMSD stabilized at ~ 0.25 nm, while the FAD ligand RMSD remained below 0.15 nm (Fig. 9A). Residue-level fluctuations at the binding site were suppressed (RMSF < 0.2 nm, Fig. 9B), indicating enhanced local rigidity upon FAD binding. The complex maintained overall compactness, with the radius of gyration (Rg) converging to 2.6–2.7 nm (Fig. 9C). The binding interface maintained 4–5 stable hydrogen bonds throughout the simulation (Fig. 9D), further supporting the complex’s high stability. The free energy landscape revealed a single deep minimum (RMSD ≈ 0.2 nm, Rg ≈ 2.7 nm; Fig. 9E), with surrounding energy barriers exceeding 18 kJ/mol, indicating a thermodynamically stable, kinetically trapped state. Collectively, these results demonstrate that FAD forms a highly stable complex with ACOX2, with the active site rigidified through persistent interactions.

## Discussion

IgA nephropathy (IgAN) is the most prevalent primary glomerulonephritis, characterized by the deposition of IgA immune complexes in the renal glomeruli, leading to progressive renal dysfunction [29, 30]. As a major cause of end-stage renal disease, IgAN imposes a substantial burden on both patients and healthcare systems [31]. Currently, the diagnosis of IgAN relies on invasive kidney biopsy, which is unsuitable for routine monitoring and lacks reliable early diagnostic biomarkers. Recent research has highlighted the critical role of cholesterol metabolism in kidney disease progression, particularly through its effects on immune modulation and inflammatory pathways [32, 33]. However, the involvement of cholesterol metabolism in the pathogenesis of IgAN remains poorly understood. This study, employing a multi-omics approach combining SMR, MR, colocalization analysis, and single-cell analysis, provided novel insights into the role of cholesterol metabolism in IgAN, offering potential biomarkers and therapeutic targets for early diagnosis and personalized treatment strategies for this disease.

In this study, SMR analysis identified four key cholesterol metabolism-related genes (ACOX2, ADAR, HPD, and MBTPS1), with their expression levels significantly associated with IgAN risk. However, Mendelian randomization (MR), colocalization analysis, and external data validation ultimately highlighted ACOX2 as the most critical gene in IgAN, positioning it as a potential biomarker for the disease. The SMR results suggest that both elevated ACOX2 expression and increased methylation levels may reduce the risk of IgAN, implying that genetic variations could modulate ACOX2 expression through methylation, thereby exerting a protective effect. MR analysis further supported the association of ACOX2 with a reduced risk of IgAN, reinforcing its role as a protective gene. Colocalization analysis demonstrated a posterior probability greater than 0.8, indicating a strong colocalization relationship between ACOX2 and IgAN, suggesting that ACOX2 plays a pivotal role in the pathogenesis of IgAN. Taken together, these findings underscore ACOX2 as a key gene involved in cholesterol metabolism and highlight its potential as a promising biological marker for IgAN, offering valuable insights for disease diagnosis and therapeutic strategies.

ACOX2 (Acyl-CoA Oxidase 2) encodes a branched-chain acyl-CoA oxidase involved in bile acid metabolism, specifically converting C27 bile acid intermediates into C24 bile acids during cholesterol processing [34, 35]. It is mainly expressed in kidney and liver [34]. ACOX2 deficiency has been linked to metabolic disorders like liver fibrosis, cerebellar ataxia, and cognitive impairment [35]. Additionally, ACOX2 functions as a tumor suppressor in hepatocellular carcinoma by inhibiting cell growth through the PPARα pathway [36]. In IgAN, ACOX2 may impact disease progression by regulating lipid metabolism and oxidative stress. Given the chronic inflammation and metabolic dysregulation in IgAN, ACOX2’s role in bile acid metabolism could modulate immune pathways, including PPARα activation, thereby attenuating inflammation. Its involvement in controlling oxidative stress and immune responses may further contribute to IgAN pathogenesis, and bile acid metabolites have been suggested to regulate the renal immune microenvironment [37, 38]. Notably, ACOX2 is predominantly expressed in proximal tubular (PT) cells, consistent with clinical observations that tubular involvement is a key determinant of renal function decline in IgAN [39]. Mesangial injury likely induces metabolic and inflammatory stress in downstream PT cells via “glomerulotubular” crosstalk, suppressing ACOX2 and disrupting lipid metabolism [40]. Consequently, altered PT ACOX2 reflects secondary metabolic remodeling from glomerular injury and may serve as a sensitive biomarker for monitoring disease progression in IgAN. Further studies are required to clarify its precise role and explore its potential as a target for therapeutic intervention.

In GSEA, ACOX2 was found to be enriched in the PI3K-Akt signaling pathway, which is involved in cell growth, survival, and metabolism [41]. This pathway plays a critical role in many diseases, including cancer [42] and immune-related kidney diseases such as IgAN [43]. In IgAN, dysregulation of the PI3K-Akt pathway may contribute to mesangial cell proliferation, matrix expansion, and inflammation. Research has suggested that PI3K-Akt signaling activation could worsen kidney damage by promoting inflammation and fibrosis in glomerular cells [44–46]. Inhibition of the PI3K/Akt/mTOR pathway has been reported to reduce cell proliferation and fibrosis, making it a potential therapeutic target for IgAN. This pathway regulates both inflammation and cell survival, key factors that drive IgAN progression. Studies in renal cells have demonstrated that PI3K-Akt signaling promotes mesangial cell proliferation and matrix production, potentially leading to glomerulosclerosis, a hallmark of advanced IgAN [47, 48]. In conclusion, ACOX2 may play a critical role in IgAN by modulating the PI3K-Akt pathway. Targeting ACOX2 could offer a promising strategy to slow or halt IgAN progression.

Based on the hub gene ACOX2, we conducted drug prediction and molecular docking analysis, identifying flavin adenine dinucleotide (FAD) as a potential therapeutic drug for IgAN. FAD is a critical coenzyme widely involved in intracellular redox reactions and metabolic regulation, particularly in the electron transport chain and lipid metabolism [49, 50]. Studies have shown that FAD possesses significant anti-inflammatory and antioxidant properties [51]. Its PEGylated complex (FAD-PEG) can significantly reduce levels of inflammatory factors such as IL-6 and TNF-α, alleviating inflammation while modulating oxidative stress to decrease reactive oxygen species (ROS) production [52]. Additionally, FAD demonstrates potential to regulate immune function and improve metabolic homeostasis [53, 54]. Regarding IgAN, which is characterized by chronic inflammation and oxidative stress, FAD may exert therapeutic effects by mitigating inflammation, reducing oxidative stress, and enhancing renal metabolic stability. Meanwhile, we performed molecular dynamics simulations, and the results showed that the ACOX2–FAD complex maintained stable binding, with RMSD stabilizing around 0.25 nm, the radius of gyration remaining consistent, and persistent hydrogen bonds throughout the trajectory. These characteristics highlight FAD as a promising candidate for IgAN treatment, although further research is needed to elucidate its underlying mechanisms.

This study highlights potential links between cholesterol metabolism and IgAN but has several limitations. The validity of MR analyses relies on core instrumental variable assumptions; although rigorous SNP selection and sensitivity analyses were performed, horizontal pleiotropy or residual confounding cannot be fully excluded. In addition, some of the data were derived from publicly available databases, which may introduce measurement error or bias. To address this limitation, we incorporated quantitative real-time PCR validation using clinical samples, providing experimental support for the bioinformatics findings. Nevertheless, the experimental sample size was relatively limited, and larger-scale in vitro and in vivo studies are warranted to further substantiate these results and elucidate the underlying mechanisms.

## Conclusion

In conclusion, our study identified ACOX2 as a key gene associated with IgAN progression. Multi-omics analyses consistently demonstrated a significant association between genetically predicted ACOX2 expression and reduced IgAN risk. Bayesian colocalization supported that these associations were driven by shared causal variants. Additionally, FAD was identified as a potential pharmacological candidate targeting ACOX2-related pathways. These findings provide new insights into the molecular mechanisms underlying IgAN and suggest that targeting ACOX2 could serve as a promising strategy for the development of personalized treatments for IgAN.

## Supplementary Information

Supplementary Material 1.

Supplementary Material 2.

Supplementary Material 3.

Supplementary Material 4.

Supplementary Material 5.

## Data Availability

The datasets supporting the conclusions of this article are available in the GeneCards database at https://www.genecards.org/, the FinnGen study at https://www.finngen.fi/, the GWAS Catalog at https://www.ebi.ac.uk/gwas/, the NCBI Gene Expression Omnibus, GEO at [https://www.ncbi.nlm.nih.gov/geo](https:/www.ncbi.nlm.nih.gov/geo).
